# Supplementary material for: Artificial intelligence applied to bed regulation in Rio Grande do Norte: Data analysis and application of machine learning on the “RegulaRN Leitos Gerais” platform
Source: PLoS One. 2024 Dec 30;19(12):e0315379. doi: 10.1371/journal.pone.0315379 (PMC11684685; doi:10.1371/journal.pone.0315379)
Supplement: Attachment — Submitted filename: renamed_025d2.pdf [file pone.0315379.s002.pdf]

PLOS ONE Reviewers' Considerations

<table>
<tr><td colspan="2">Reviewer 1:</td></tr>
<tr><td>This study effectively applies machine learning to the RegulaRN Leitos Gerais platform to optimize hospital bed regulation in Rio Grande do Norte. Analyzing data from October 2021 to January 2024, it shows strong performance from models like XGBoost, Random Forest, and Gradient Boosting in accuracy, precision, and recall. However, the study would benefit from incorporating additional methods such as k-Nearest Neighbors (kNN) and Support Vector Machines (SVM), as well as referencing recent literature (e.g., doi.org/10.1109/ACCESS.2024.3392729 and doi.org/10.1016/j.patcog.2023.109641) to ensure a comprehensive evaluation and alignment with current advancements in healthcare machine learning.</td><td>Dear reviewer, we appreciate your important comments. We truly understand that using other computational methods would enable greater gains for the study. However, these models listed by the reviewer are not part of the initial scope of the study. In this context, we understand that this was a limitation of the study, for this reason we highlighted this limitation in the manuscript in the “Conclusion” section and justified it using the references recommended by the reviewer. We also describe that the use of these computational methods recommended by the reviewer may be used in future work.</td></tr>
<tr><td colspan="2">Reviewer 2:</td></tr>
<tr><td>Suggested Improvement: The introduction clearly outlines the general problem of bed regulation but could be more detailed in discussing the relevance of artificial intelligence in this specific context by comparing it with similar studies.
o Lines 60-61: "Intelligent computer models can help reduce the impact of uncertainties and ambiguities in the regulatory process and improve decision-making support."
o Replace with: "Intelligent computer models have demonstrated significant potential in healthcare systems by reducing uncertainties and ambiguities in complex decision-making processes. For example, prior studies in similar healthcare contexts have shown that machine learning models can enhance hospital management by optimizing resource allocation and reducing patient waiting times (reference). This study aims to build on these findings to demonstrate the effectiveness of AI-based models specifically in bed regulation in Rio Grande do Norte."</td><td>Dear reviewer, we appreciate your important comments, as they contributed greatly to improving the final quality of the manuscript, thank you very much. Therefore, all recommendations were duly met.</td></tr>
<tr><td>More detailed discussion on model</td><td>Dear reviewer, we appreciate your</td></tr>
</table>

limitations The article mentions limitations but could provide a more robust discussion of how these limitations affect the results and suggest strategies to mitigate them.<br>• Line 398: "The study’s limitations include the fact that the health professionals did not provide more precise information on some of the data that could have been better analyzed in the models, such as whether the patient was pregnant."<br>• Replace with: "A significant limitation of this study is the incomplete dataset, particularly the absence of detailed information such as pregnancy status and gestational age. This missing information could introduce bias in model predictions, particularly for patient subgroups with different clinical needs. Future work should focus on improving data collection protocols to ensure that such critical variables are recorded, allowing for more nuanced and accurate model predictions across diverse patient groups."	important comments, as they contributed greatly to improving the final quality of the manuscript, thank you very much. Therefore, all recommendations were duly met.
More detailed explanation of the choice of machine learning models The choice of machine learning models is explained, but a more in-depth justification of why certain models (like XGBoost and Random Forest) were selected would be helpful.<br>• Line 165: "The definition of models for data classification involved algorithms that, according to the literature, perform well with high volumes of data."<br>• Replace with: "The selection of classification models was based on their proven ability to handle large volumes of imbalanced healthcare data. XGBoost, for example, has been shown to perform well in healthcare settings due to its gradient boosting framework, which effectively handles missing data and provides robust performance on tabular datasets (Chen & Guestrin, 2016). Random Forest, on the other hand, was selected for its ability to manage complex decision trees and its resistance to overfitting, particularly in high-dimensional datasets (Breiman, 2001). These models were chosen for their complementarity in addressing the specific challenges of bed regulation data in this study."	Dear reviewer, we appreciate your important comments, as they contributed greatly to improving the final quality of the manuscript, thank you very much. Therefore, all recommendations were duly met.
Better organization of tables and figures	Dear reviewer, we appreciate your

The presentation of tables and figures can be improved with more descriptive captions and the inclusion of an analysis immediately after presenting each figure/table to facilitate the interpretation of the results.<br>• Line 276: "Table 6 shows all the values obtained."<br>• Replace with: "Table 6 presents the performance metrics for each machine learning model, including accuracy, precision, recall, F1-Score, and specificity. Notably, XGBoost outperformed the other models in accuracy and recall, making it a robust choice for predicting patient outcomes in bed regulation. However, the high specificity observed in the MLP models indicates that these models may be more suitable when the goal is to minimize false positives, particularly in critical care cases."	important comments, as they contributed greatly to improving the final quality of the manuscript, thank you very much. Therefore, all recommendations were duly met.
More in-depth discussion of the practical impact of the results The discussion could delve deeper into the practical impact of adopting AI in the healthcare system and the challenges of implementing it on a large scale.<br>• Line 349: "At the management level, in response to a more efficient regulatory system, financial and human resources can be distributed in a way that is more coherent with the needs of the different health sectors."<br>• Replace with: "At the management level, adopting AI-driven systems for bed regulation could lead to significant improvements in resource allocation, reducing patient wait times and optimizing bed occupancy rates. However, implementing these systems at scale presents challenges, such as ensuring adequate training for health professionals and integrating AI tools with existing hospital infrastructure. Addressing these challenges will be critical for maximizing the potential benefits of AI in the public healthcare system."	Dear reviewer, we appreciate your important comments, as they contributed greatly to improving the final quality of the manuscript, thank you very much. Therefore, all recommendations were duly met.
I noticed the absence of graphs and visual figures that could significantly improve the clarity and understanding of the article, especially concerning the methodology and results. The inclusion of graphical elements such as flowcharts and result charts would be a valuable contribution to make the	Dear reviewer, we appreciate your important comments, as they contributed greatly to improving the final quality of the manuscript, thank you very much. Therefore, all recommendations were duly met.

methodological process more accessible and the data easier to interpret.<br>Firstly, presenting a detailed methodology flowchart would be extremely helpful in guiding readers through all the stages described in the article. This would clarify the process from data extraction and processing to machine learning model selection, allowing for a clearer understanding of the methodological sequence.<br>Additionally, to facilitate the understanding of the results, it would be interesting to include graphs comparing the performance metrics of the different machine learning models. Bar graphs, for example, could visually show the accuracy, recall, specificity, and F1-Score of the tested models. The inclusion of ROC-AUC curves would also help visually present each model's discriminative capabilities, making the information easier to interpret for readers.<br>Finally, the article mentions correlation analysis between variables, but including a correlation matrix, such as a heatmap, could visually highlight the most significant relationships between the variables. This type of graphical visualization would make data interpretation more immediate and intuitive for the reader.<br>These visual additions, such as flowcharts and result charts, would not only enhance the manuscript's clarity but also help more effectively convey the complexities involved in data analysis and the results obtained.	
Reviewer 3	
The text presents clear data, and the need for minor adjustments indicated in the submitted manuscript. Duplicate publication, whether intentional or not, may harm the credibility of the research and compromise the intellectual property rights of both journals.	Dear reviewer, thank you very much for your comments, they were valuable for improving our manuscript.<br><br>Regarding the adjustments, I would like to inform you that they were duly made at several points in the manuscript. Thank you very much for this recommendation, as it caught my attention to review and improve the text once again.<br><br>Regarding the duplicate publication, I would like to inform you that these are manuscripts with completely different

	research objects. One deals with COVID-19 and the other with patients who were admitted to general hospital beds, that is, for any other reason.<br><br>The already published manuscript, despite working with data from the RegulaRN Platform, which is the same as this manuscript under review, deals specifically with analyses performed on data from COVID-19 patients. Therefore, these are different data from those used in this manuscript still under review.<br><br>Another point that greatly differentiates the manuscripts is the computational models used. Therefore, both the methodology and the results presented are quite different.<br><br>There can be no duplication of publications, since the first manuscript deals with patients with COVID-19 admitted to hospital beds during the COVID-19 pandemic and the article under review deals with data from patients who were admitted for any other reason, including the data not from the COVID-19 pandemic period - hence general beds. I hope I have clarified the topic.<br><br>I would like to thank the reviewer for the opportunity to clarify. I am available for any further questions.
