# Supplementary material for: Artificial intelligence applied to bed regulation in Rio Grande do Norte: Data analysis and application of machine learning on the “RegulaRN Leitos Gerais” platform
Source: PLoS One. 2024 Dec 30;19(12):e0315379. doi: 10.1371/journal.pone.0315379 (PMC11684685; doi:10.1371/journal.pone.0315379)
Supplement: Attachment — Submitted filename: PONE-D-24-27498_reviewer.docx [file pone.0315379.s001.docx]

PLOS ONE

# Artificial Intelligence Applied to Bed Regulation in Rio Grande do Norte: Data Analysis and Application of Machine Learning on the “RegulaRN Leitos Gerais” Platform

--Manuscript Draft--

| **Manuscript Number:** | PONE-D-24-27498 |
| --- | --- |
| **Article Type:** | Research Article |
| **Full Title:** | Artificial Intelligence Applied to Bed Regulation in Rio Grande do Norte: Data Analysis and Application of Machine Learning on the “RegulaRN Leitos Gerais” Platform |
| **Short Title:** | Artificial Intelligence Applied to Bed Regulation in Rio Grande do Norte |
| **Corresponding Author:** | Tiago Barreto  Federal University of Rio Grande do Norte: Universidade Federal do Rio Grande do Norte  Natal, BRAZIL |
| **Keywords:** | Machine Learning; Computational Methods; Bed Regulation, RegulaRN Leitos Gerais. |
| **Abstract:** | Bed regulation within Brazil's National Health System (SUS) plays a crucial role in managing care for patients in need of hospitalization. In Rio Grande do Norte, Brazil, the RegulaRN Leitos Gerais platform was the information system developed to register requests for bed regulation for COVID-19 cases. However, the platform was expanded to cover a range of diseases that require hospitalization. This study explored different machine learning models in the RegulaRN database, from October 2021 to January 2024, totaling 47,056 regulations. From the data obtained, 12 features were selected from the 24 available. After that, blank and inconclusive data were removed, as well as the outcomes that had values other than discharge and death, rendering a binary classification. Data was also correlated, balanced, and divided into training and test portions for application in machine learning models. The results showed better accuracy (87.77\%) and recall (87.77\%) for the XGBoost model, and higher precision (87.85\%) and F1-Score (87.56\%) for the Random Forest and Gradient Boosting models, respectively. As for Specificity (82.94\%) and ROC-AUC (82.13\%), the Multilayer Perceptron with SGD optimizer obtained the highest scores. The results evidenced which models could adequately assist medical regulators during the decision-making process for bed regulation, enabling even more effective regulation and, consequently, greater availability of beds and a decrease in waiting time for patients. |
| **Order of Authors:** | Tiago de Oliveira Barreto |
|  | Fernando Lucas de Oliveira Farias |
|  | Nicolas Vinícius Rodrigues Veras |
|  | Pablo Holanda Cardoso |
|  | Gleyson José Pinheiro Caldeira Silva |
|  | Chander de Oliveira Pinheiro |
|  | Maria Valéria Bezerra Medina |
|  | Felipe Ricardo dos Santos Fernandes |
|  | Ingridy Marina Pierre Barbalho |
|  | Lyane Ramalho Cortez |
|  | João Paulo Queiroz dos Santos |
|  | Antonio Higor Freire de Morais |
|  | Gustavo Fontoura de Souza |
|  | Guilherme Medeiros Machado |
|  |  |

|  | Márcia Jacyntha Nunes Rodrigues Lucena |
| --- | --- |
|  | Ricardo Alexsandro de Medeiros Valentim |
| **Additional Information:** |  |
| **Question** | **Response** |
| **Financial Disclosure**  Enter a financial disclosure statement that describes the sources of funding for the work included in this submission. Review the [submission guidelines](http://journals.plos.org/plosone/s/submission-guidelines#loc-financial-disclosure-statement) for detailed requirements. View published research articles from [*PLOS ONE*](https://journals.plos.org/plosone/) for specific examples.  This statement is required for submission and **will appear in the published article** if the submission is accepted. Please make sure it is accurate. | Yes |
| **Funded studies**  Enter a statement with the following details:   - Initials of the authors who received each award - Grant numbers awarded to each author - The full name of each funder - URL of each funder website - Did the sponsors or funders play any role in the study design, data collection and analysis, decision to publish, or preparation of the manuscript? |  |
| Did you receive funding for this work? |  |
| Please add funding details.  as follow-up to "**Financial Disclosure**  Enter a financial disclosure statement that describes the sources of funding for the work included in this submission. Review the [submission guidelines](http://journals.plos.org/plosone/s/submission-guidelines#loc-financial-disclosure-statement) for detailed requirements. View published research articles from [*PLOS ONE*](https://journals.plos.org/plosone/) for specific examples.  This statement is required for submission and **will appear in the published article** if the submission is accepted. Please make | The present study was funded through the Project Regula SESAP-RN/FUNCERN, grant number 69/2021, carried out by the Laboratory of Technological Innovation in Health (LAIS) of the Federal University of Rio Grande do Norte (UFRN) in cooperation with the Secretary of Public Health of Rio Grande do Norte. |

| sure it is accurate. |  |
| --- | --- |
| **Funded studies**  Enter a statement with the following details:   - Initials of the authors who received each award - Grant numbers awarded to each author - The full name of each funder - URL of each funder website - Did the sponsors or funders play any role in the study design, data collection and analysis, decision to publish, or preparation of the manuscript? |  |
| Did you receive funding for this work?" |  |
| Please select the country of your main research funder (please select carefully as in some cases this is used in fee calculation).  as follow-up to "**Financial Disclosure**  Enter a financial disclosure statement that describes the sources of funding for the work included in this submission. Review the [submission guidelines](http://journals.plos.org/plosone/s/submission-guidelines#loc-financial-disclosure-statement) for detailed requirements. View published research articles from [*PLOS ONE*](https://journals.plos.org/plosone/) for specific examples.  This statement is required for submission and **will appear in the published article** if the submission is accepted. Please make sure it is accurate. | BRAZIL - BR |
| **Funded studies**  Enter a statement with the following details:   - Initials of the authors who received each award - Grant numbers awarded to each author - The full name of each funder - URL of each funder website - Did the sponsors or funders play any role in the study design, data collection and analysis, decision to publish, or preparation of the manuscript? |  |
| Did you receive funding for this work?" |  |

| **Competing Interests**  Use the instructions below to enter a competing interest statement for this submission. On behalf of all authors, disclose any [competing interests](http://journals.plos.org/plosone/s/competing-interests) that could be perceived to bias this work—acknowledging all financial support and any other relevant financial or non- financial competing interests.  This statement is required for submission and **will appear in the published article** if the submission is accepted. Please make sure it is accurate and that any funding sources listed in your Funding Information later in the submission form are also declared in your Financial Disclosure statement.  View published research articles from  [*PLOS ONE*](http://journals.plos.org/plosone/) for specific examples. | The authors have declared that no competing interests exist. |
| --- | --- |
| **NO authors have competing interests**  Enter: *The authors have declared that no competing interests exist.*  **Authors with competing interests**  Enter competing interest details beginning with this statement:  *I have read the journal's policy and the authors of this manuscript have the following competing interests: [insert competing interests here]* |  |
| * typeset |  |
| **Ethics Statement**  Enter an ethics statement for this submission. This statement is required if the study involved:   - Human participants - Human specimens or tissue - Vertebrate animals or cephalopods | N/A |

| - Vertebrate embryos or tissues - Field research   Write "N/A" if the submission does not require an ethics statement.  General guidance is provided below. Consult the [submission guidelines](http://journals.plos.org/plosone/s/submission-guidelines#loc-guidelines-for-specific-study-types) for detailed instructions. **Make sure that all information entered here is included in the Methods section of the manuscript.** |  |
| --- | --- |

| **Format for specific study types**  **Human Subject Research (involving human participants and/or tissue)**   - Give the name of the institutional review board or ethics committee that approved the study - Include the approval number and/or a statement indicating approval of this research - Indicate the form of consent obtained (written/oral) or the reason that consent was not obtained (e.g. the data were analyzed anonymously)   **Animal Research (involving vertebrate animals, embryos or tissues)**   - Provide the name of the Institutional Animal Care and Use Committee (IACUC) or other relevant ethics board that reviewed the study protocol, and indicate whether they approved this research or granted a formal waiver of ethical approval - Include an approval number if one was obtained - If the study involved *non-human primates*, add *additional details* about animal welfare and steps taken to ameliorate suffering - If anesthesia, euthanasia, or any kind of animal sacrifice is part of the study, include briefly which substances and/or methods were applied   **Field Research**  Include the following details if this study involves the collection of plant, animal, or other materials from a natural setting:   - Field permit number - Name of the institution or relevant body that granted permission |  |
| --- | --- |
|  |  |
| **Data Availability**  Authors are required to make all data underlying the findings described fully available, without restriction, and from the time of publication. PLOS allows rare exceptions to address legal and ethical concerns. See the [PLOS Data Policy](http://journals.plos.org/plosone/s/data-availability) and [FAQ](http://journals.plos.org/plosone/s/data-availability#loc-faqs-for-data-policy) for detailed information. | Yes - all data are fully available without restriction |

| A Data Availability Statement describing where the data can be found is required at submission. Your answers to this question constitute the Data Availability Statement and **will be published in the article**, if accepted. |  |
| --- | --- |
| **Important:** Stating ‘data available on request from the author’ is not sufficient. If your data are only available upon request, select ‘No’ for the first question and explain your exceptional situation in the text box. |  |
| Do the authors confirm that all data underlying the findings described in their manuscript are fully available without restriction? |  |
| **Describe where the data may be found in full sentences. If you are copying our sample text, replace any instances of XXX with the appropriate details.**   - If the data are **held or will be held in a public repository**, include URLs, accession numbers or DOIs. If this information will only be available after acceptance, indicate this by ticking the box below. For example: *All XXX files are available from the XXX database (accession number(s) XXX, XXX.*). - If the data are all contained **within the manuscript and/or Supporting Information files**, enter the following: *All relevant data are within the manuscript and its Supporting Information files.* - If neither of these applies but you are able to provide **details of access elsewhere**, with or without limitations, please do so. For example:   *Data cannot be shared publicly because of [XXX]. Data are available from the XXX Institutional Data Access / Ethics Committee (contact via XXX) for researchers who meet the criteria for access to confidential data.*  *The data underlying the results presented in the study are available from (include the name of the third party* | The data used in this research can be accessed via the link: https://zenodo.org/records/11387710 |

| *and contact information or URL).*   - This text is appropriate if the data are owned by a third party and authors do not have permission to share the data.   * typeset |  |
| --- | --- |
| Additional data availability information: |  |

Manuscript	[Click here to](https://www2.cloud.editorialmanager.com/pone/download.aspx?id=36821430&guid=fac1b7a8-be6b-4c9c-b377-02ff9494bc89&scheme=1)

[access/download;Manuscript;plos_latex_template_v3_6 (1).pdf](https://www2.cloud.editorialmanager.com/pone/download.aspx?id=36821430&guid=fac1b7a8-be6b-4c9c-b377-02ff9494bc89&scheme=1)

Artificial Intelligence Applied to Bed Regulation in Rio Grande do Norte: Data Analysis and Application of Machine Learning on the “RegulaRN Leitos Gerais” Platform

Tiago de Oliveira Barreto^1*^, Fernando Lucas de Oliveira Farias ^1^, Nicolas Vin´ıcius Rodrigues Veras^1,2^, Pablo Holanda Cardoso^1,2^, Gleyson Jos´e Pinheiro Caldeira Silva^1^, Chander de Oliveira Pinheiro^3^, Maria Val´eria Bezerra Medina^3^, Felipe Ricardo dos Santos Fernandes^1^, Ingridy Marina Pierre Barbalho^1^, Lyane Ramalho Cortez^1,3^, Jo˜ao Paulo Queiroz dos Santos^1,2^, Antonio Higor Freire de Morais^1,2^, Gustavo Fontoura de Souza^1,2^, Guilherme Medeiros Machado^4^, M´arcia Jacyntha Nunes Rodrigues Lucena^5^, Ricardo Alexsandro de Medeiros Valentim^1^,

1 Laboratory of Technological Innovation in Health (LAIS), Federal University of Rio Grande do Norte (UFRN), Natal, Rio Grande do Norte, Brazil
2 Advanced Nucleus of Technological Innovation (NAVI), Federal Institute of Rio Grande do Norte (IFRN), Natal, Rio Grande do Norte, Brazil
3 Secretary of Public Health of Rio Grande do Norte, Natal, Rio Grande do Norte, Brazil
4 LyRIDS, ECE-Engineering School, Paris, France
5 Department of Informatics and Applied Mathematics, Federal University of Rio Grande do Norte (UFRN), Natal, Rio Grande do Norte, Brazil

* [tiago.barreto@lais.huol.ufrn.br](mailto:tiago.barreto@lais.huol.ufrn.br)

**Abstract**

Bed regulation within Brazil’s National Health System (SUS) plays a crucial role in managing care for patients in need of hospitalization. In Rio Grande do Norte, Brazil, the *RegulaRN Leitos Gerais* platform was the information system developed to register requests for bed regulation for COVID-19 cases. However, the platform was expanded to cover a range of diseases that require hospitalization. This study explored different machine learning models in the RegulaRN database, from October 2021 to January 2024, totaling 47,056 regulations. From the data obtained, 12 features were selected from the 24 available. After that, blank and inconclusive data were removed, as well as the outcomes that had values other than discharge and death, rendering a binary classification. Data was also correlated, balanced, and divided into training and test portions for application in machine learning models. The results showed better accuracy (87.77%) and recall (87.77%) for the XGBoost model, and higher precision (87.85%) and F1-Score (87.56%) for the Random Forest and Gradient Boosting models, respectively. As for Specificity (82.94%) and ROC-AUC (82.13%), the Multilayer Perceptron with SGD optimizer obtained the highest scores. The results evidenced which models could adequately assist medical regulators during the decision-making process for bed regulation, enabling even more effective regulation and, consequently, greater availability of beds and a decrease in waiting time for patients.

# Introduction 1

In Brazil, the hospital bed regulation process of the Brazilian Health System (SUS)	2

plays a fundamental role in the management and distribution of care for patients	3

requiring hospitalization [1, 2]. However, although the National Regulation Policy was	4

instituted more than 15 years ago by Ordinance No. 1,559 of August 2008 [3] and	5

consolidated by Ordinance No. 02 of September 2017 (Brazil, 2017) [4], many regions	6

have difficulties in ensuring correct regulatory conduct.	7

In this context, in addition to organizational issues, the regulation system in Brazil	8

still faces recurring problems such as the precariousness of hospital infrastructure,	9

overcrowding in health units, an insufficient number of beds, difficulties in integration	10

and communication between the entities involved in the regulatory process, greater	11

transparency in processes and allocation of resources, in addition to not having efficient	12

systems to help the regulation process [1, 5]. In Brazil, due to a non-mandatory	13

recommendation from the Ministry of Health (MoH), the Regulation System - SISREG -	14

is still used in many Brazilian states. This system was created in 2001 and is made	15

available by the Brazilian Health System Informatics Department (DATASUS) [6].	16

Currently, this system is considered obsolete and inadequate, especially due to the lack	17

of interoperability with the SUS technological ecosystem itself and the lack of	18

transparency [7]. This is a legacy health information system which, although it is still	19

used, is no longer able to play an effective role in the National Policy for the Regulation	20

of Assistance in Access to Health Services in Brazil.	21

Until April 2020, the center for regulating access to health services in the state of	22

Rio Grande do Norte did not have a platform for regulating hospital beds to	23

systematically organize regulatory conduct within the scope of the SUS in the state.	24

The regulatory flow control measures used were based on spreadsheets, e-mails and	25

telephone communication, and messaging systems [8, 9].	26

Faced with the serious public health crisis caused by the COVID-19 pandemic, the	27

government of the state of Rio Grande do Norte has set up technical-scientific	28

cooperation between researchers in the field of digital health and the managers and	29

formulators of public health policies at the State Secretariat of Public Health of RN	30

(SESAP/RN). The aim of this technical-scientific cooperation was to formulate and	31

implement a digital health solution that would make it possible to control and monitor	32

the entire process of regulating hospital beds in all the state’s public hospitals online, on	33

time and transparently, totaling 24 public hospitals, with more than 900 beds available.	34

Based on this technical-scientific cooperation, the RegulaRN Platform for COVID-19	35

was developed and implemented throughout the state of Rio Grande do Norte, whose	36

initial objective was to monitor and control access to hospital beds in wards and	37

intensive care units (ICUs) for the disease during the pandemic [2, 8, 9]. The state of Rio	38

Grande Norte, which is located in the northeastern region of Brazil, currently has an	39

area of 52,797 km² and a population of approximately 3.5 million inhabitants.	40

After the implementation of the RegulaRN Platform, it became necessary to expand	41

the digital health solution to the other regulatory specialties. The system is currently	42

responsible for regulating access to beds, vascular surgery, outpatient care, exams, and	43

consultations. In this way, the RegulaRN Platform has become a unique digital health	44

solution for the management of health regulation services in the state of Rio Grande do	45

Norte, an important aspect because it has centralized and integrated, through	46

international interoperability standards, the Health Data Network (RDS) with all the	47

other technologies in the state’s public health ecosystem that are necessary for the	48

process of regulating access to health services.	49

The health regulation process needs to be carried out in a rigorous, agile and	50

transparent manner, as the incorrect conduct of a regulatory process in public health	51

has intrinsic impacts on waiting times for access to hospital beds, as well as on	52

hospitalization times, which can have negative impacts on the availability of hospital	53

beds and increase the potential for existing problems [5, 10]. In this way, the inefficiency	54

and ineffectiveness of this process can aggravate public health crisis situations, such as	55

the COVID-19 pandemic, as it requires more rational use of health resources [8, 11–16].	56

Therefore, due to its complexity and the pressures that exist in all segments of the	57

regulatory process, investment in intelligent computer systems can maximize the correct	58

direction and assertive decision-making in healthcare systems [17–22].	59

Intelligent computer models can help reduce the impact of uncertainties and	60

ambiguities in the regulatory process and improve decision-making support [23, 24]. As	61

for the process of evaluating hospital outcomes, several studies have used machine	62

learning models to help with their predictions [25–30].	63

In this context, the aim of this work is to analyze data from the *RegulaRN Leitos*	64

*Gerais* Platform and use it to train and validate different machine learning models.	65

Subsequently, to choose the most significant classification model capable of predicting	66

the outcome of patients regulated by the *RegulaRN Leitos Gerais* platform with greater	67

accuracy, precision, recall, specificity, F1 Score, and ROC-AUC. Furthermore, discuss	68

the main impacts and potential of a digital health solution on the decision-making	69

process of regulatory professionals.	70

# Materials and methods 71

The methodological bias of this paper consists in two main steps: exploratory data	72

analysis and applying the data to computer models. In the evaluation process, the data	73

was extracted, evaluated, characterized, pre-processed, and correlated. For the	74

application stage, concerning the computational models, four phases were taken into	75

account: 1) definition of evaluation metrics; 2) data balancing and division into training	76

and validation groups; 3) selecting the models for classification, and 4) hyperparameters	77

to choose the best performing model; in line with Barreto et al [2].	78

**Extraction, evaluation, characterization and pre-processing**	79

This study used the database from the RegulaRN Leitos Gerais platform, a system	80

adopted to manage the regulation flow of SUS beds in the state of Rio Grande do Norte.	81

The database covers the period of October 2021 to January 2024, with 47.056	82

regulations in the two-state centers (Metropolitan and West). From this total, 1,868	83

regulations were removed because they were linked to newborn regulations, and these	84

have different clinical assessment protocols when compared to adult and pediatric	85

patients. The initial analysis therefore included 45,188 regulation requests. A more	86

detailed descriptive analysis of the data is presented in the results section.	87

The initial data extraction included 24 features: a) date of request; b) occupancy	88

type; c) case type; d) unified prioritization score (EUP); e) Sequential Organ Failure	89

Assessment (SOFA) scale; f) type of hospital bed requested; g) admission date; h) type	90

of input bed; i) discharge date; j) discharge bed type; k) national health card number; l)	91

gender; m) patient’s municipality; n) patient’s federative unit; o) pregnant woman (yes	92

or no); p) gestational period; q) age; r) regulator identification; s) outcome; t)	93

requesting health unit; u) municipality of the requesting health unit; v) providing health	94

unit; w) municipality of the providing health unit and x) ICD.	95

Thus, the features that were not associated with the patient’s clinical condition and	96

do not show any impact in the final result, or that relate to the locality record, such as:	97

date of request, national health card number, patient’s federative unit, patient’s	98

municipality, regulator identification, requesting health unit, municipality of the	99

requesting health unit, and municipality of the providing health unit. In addition,	100

features with only one possible record or insufficient information were also removed:	101

type of occupation, type of case, pregnant woman (yes or no), and gestational age.	102

Consequently, only 12 characteristics were selected, namely: EUP score, SOFA scale,	103

type of hospital bed requested, admission date, admission bed type, discharge date,	104

discharge bed type, gender, age, outcome, providing health unit, and ICD. Using the	105

entry date and exit date features, it was possible to create the patient’s hospitalization	106

time feature. As a result, 11 features were used in the classification process. Table 1	107

shows the description of all the data types extracted from RegulaRN Leitos Gerais.	108

After extracting the data, we evaluated the values contained in all the features and	109

in order to guarantee the integrity of the analysis, the lines with blank data or	110

inconclusive information were removed. In addition, the target column “outcome”	111

contained six different values, namely: by discharge, by death, for other reasons, by stay,	112

by delivery procedure, by transfer, etc. As these last four outcomes do not properly	113

indicate a positive or negative closure of the regulation, as well as having a lower	114

number of recurrences, around 7.151 regulations were removed. This maintains a binary	115

classification (by discharge - positive, or by death - negative) for the computer models.	116

Finally, 38.023 effective regulations were selected for application in the artificial	117

intelligence models. Fig 1 shows the design used to process and select the data.	118

Furthermore, in order to enable the reproducibility of this experiment, the final database	119

used is available on the zenodo platform (https://zenodo.org/records/11387710),	120

## Fig 1. Workflow defined for data processing and selection.

**Correlation between dataset features**	121

The first task was to perform a pairwise correlation of the features. The objective is to	122

identify features with greater or lesser correspondence with others. As many of these	123

are categorical data, the phik correlation model was implemented in this analysis. Phik	124

is abble to consistently correlate variables from several backgrounds, being categorical,	125

ordinals and intervals a like, turning into a refinement of Pearson [31] hypothesis test.	126

**Definition of evaluation metrics**	127

The overall aim of the study is to classify hospital bed regulation data to predict a	128

patient’s positive or negative outcome. Furthermore, it is important to investigate the	129

models’ performance in situations where predictions are wrong, either due to a high	130

number of false positives or false negatives. Thus, it is necessary to include not only	131

accuracy, but also precision, recall, specificity, F1-Score, and ROC-AUC in a similar way	132

to those found in the works of Iwendi et al [32], Aljameel et al [33] and Endo et al [34].	133

The accuracy consists in the set of data with correct predictions (true positive and	134

true negative) divided by the sum of all predictions made by the model (true positive,	135

true negative, false positive, false negative) (Equation 1):	136

*Accuracy* = (*TP* + *TN* )*/*(*TP* + *FP* + *FN* + *TN* )	(1)

Precision consists of dividing the true positive rate by the sum of the true positive	137

and false positive rates (Equation 2).	138

*Precision* = *TP/*(*TP* + *FP* )	(2) Recall involves the rate of true positives divided by the rate of true positives plus	139

false negatives (Equation 3).	140

**Table 1.** Description of database.

## Data description

**Field	Description**

request date	Represents the date a bed request was registered.

type of occupa- tion

type of occupa- tion

Represents the type of occupation of the regulation request.

Represents the type of occupation of the regulation request.

case of type	Represents the results of tests for patients who were suspected of having Covid-

19. The results could be: positive, negative, inconclusive, or null.

Represents the EUP score value. The numerical scale ranges from 2 to 8 and

EUP score

is associated with the Charlson Comorbidity Index, Clinical Frailty Scale and simplified SOFA.

SOFA scale	Represents the patient’s prioritization value according to the values of this scale.

requested	bed type

Represents the type of bed selected by the regulation center for a patient. The results could be: ward and uci.

entry date	Represents the date the patient was allocated to the health unit (hospital). entry bed type	Represents the type of bed that the patient was allocated in the health unit

(hospital). The results could be: ward and uci.

output date	Represents the date that the patient left the bed after the outcome. output bed type	Represents the type of bed the patient was in before the outcome.

sus card number	Represents each patient’s SUS card number. Given by a 15-digit sequence. sex	Represents the patient’s sex.

patient’s munic- ipality

patient’s federal unit

Indicates the patient’s city of residence.

Is the acronym for the patient’s federal unit.

pregnant	Represents whether the patient is pregnant or not. gestational age	Describe how far along the pregnancy is, measured in weeks. age	Represents patient age.

regulator	Represents the regulator identification responsible for regulation.

outcome	Represents the outcome of the patient in bed. Possible values for this field are: discharge and death.

requesting health unit municipality of the	requesting health unit provider health unit municipality of the	provider health unit

Represents the unit health that solicits a bed for the patient.

Represents the municipality of the health unit that solicits the bed.

Represents the health unit that admits and accommodates the patient in the bed.

Represents the municipality of the health unit that receives and accomodate the patient in the bed.

ICD	Represents the International Classification of Diseases for bed regulation.

*Recall* = *TP/*(*TP* + *FN* )	(3)

Specificity refers to the prediction of true negatives divided by the sum of true	141

negatives and false positives (Equation 4).	142

*Specificity* = *TN/*(*TN* + *FP* )	(4)

The F1-score is the harmonic mean between the precision and recall. The formula	143

involves the product of precision and recall divided by the sum of these metrics,	144

multiplied by 2 (Equation 5).	145

| *F* 1*Score* = 2 ∗ (*Precision* ∗ *Recall*)*/*(*Precision* + *Recall*) | (5) |  |
| --- | --- | --- |
| ROC-AUC can be obtained by recall divided by the complementary value | of | 146 |
| specificity (Equation 6). |  | 147 |

*ROCAUC* = *Recall/*(1 − *Specificity*)	(6)

**Data balancing and splitting into training and validation data**	148

The RegulaRN Leitos Gerais Platform database refers to real-world bed regulation data,	149

in this sense, there is an unbalanced distribution of data when classified by outcome,	150

82.6% are discharges and 17.4% deaths. The use of an unbalanced database biases the	151

machine learning classifiers, making the algorithms able to identify patterns from the	152

predominant class much better than patterns from the minority class. To mitigate this	153

problem, one of the most common techniques is SMOTE (Synthetic Minority	154

Over-sampling), which works by increasing the number of data points in the minority	155

class [35]. The SMOTE algorithm first identifies the minority class, then in the feature	156

vector space identifies the k nearest neighbors of that class (k is usually equal to 5).	157

Finally, a new instance of the minority class is generated by randomly selecting values	158

in the vector space between an instance of the minority class and the nearest neighbors	159

identified. This process is repeated until the database is completely balanced.	160

In addition, as for the division of training and validation data, the same	161

segmentation was used as in other studies applying machine learning techniques that	162

use a large volume of data [36–38]. Therefore, 80% of the data was directed to training	163

and the others 20% for validation.	164

**Definition of models for data classification**	165

The definition of models for data classification involved algorithms that, according to	166

the literature, perform well with high volumes of data [37, 39–41]. Thus, Decision	167

Tree [42], Random Forest [43], Gradient Boosting [44], Adaboost [45], XGBoost [46] and	168

Multi-Layer Perceptron [47], were selected as techniques in the development of this	169

study. As for the MLP, two different paths were taken, the Stochastic Gradient Descent	170

(SGD), due to its performance, and Adam because of its consistency in treating gradient	171

explosion and fading problems [48].	172

**Hyperparameters to define the best model**	173

After the model selection, it was necessary to define the best combination of	174

hyperparameters to enhance the evaluation metrics of each model. Thus, this section	175

presents which hyperparameters were adopted and which methods were elaborated in	176

the training and validation steps. It is worth mentioning that all computational model	177

development in this research used python’s sckit-learn library [49].	178

For each selected model, hyperparameters were set aiming to boost the performance 179 metrics. In this regard, the following hyperparameters were selected for Decision Tree: 180 criterion, which measures the quality of node splitting; max depth of tree, which	181

determines the maximum depth of the tree; min samples leaf, which represents the	182

minimum number of samples needed in a leaf; and max features, which considers the	183

maximum number of features analyzed to perform a split. For the Random Forest and	184

Gradient Boosting models, the criterion, max depth of the tree and max features were	185

also used, including the number estimators, which considers the number of trees in the	186

forest. In the Adaboost model, the parameters number estimators, learning rate and	187

algorithm were chosen. The learning rate refers to the learning weight at each iteration,	188

while the algorithm relates to how the model can speed up the convergence of the	189

classifier with the least possible error. For XGBoost: learning rate, number estimators,	190

max depth and colsample by tree. This last hyperparameter is associated with the	191

randomly selected fraction of resources that will be used to train each tree. Finally, for	192

the MLP Adam and MLP SGD models, the following were used: hidden layer size,	193

which represents the number of layers in the model; activation, which represents the	194

model’s activation function and batch size, which represents the size of the minibatches	195

that will be used to help the optimizers.	196

The grid GridSearchCV functionality, which allows all possible combinations of	197

hyperparameters to be iterated, was applied during the training to find which	198

parameters showed better results in the evaluated metrics [50, 51]. A proportional	199

division of the training and test data was also carried out randomly using the cross	200

validation attribute with a value of 10-folds in the GridSearchCV functionality, as a way	201

of enhancing the model’s learning. In addition, the models were trained five times,	202

similar to that developed by Ahsan et al [52], in order to determine the best set of	203

hyperparameters more accurately. The details of the hyperparameters used and the	204

respective values chosen for each model are shown in Table 2.	205

**Results**	206

**General data analysis**	207

Considering the data profile from *RegulaRN Leitos Gerais*, between October 2021 and	208

January 2024, it was possible to identify that most hospitalizations involve male adults,	209

young people and children, in hospital beds and with lower EUP score and SOFA scale.	210

In addition, the database contains outcomes by each provider hospital (41), to	211

address which health units had the highest number of requests and their respective	212

outcomes, given that his feature showed a high correlation with several other dataset	213

variables. Each hospital has a different treatment specialty, and thus some receive	214

requests of greater complexity and mortality than others, culminating in different	215

proportions of discharges and deaths. Finally, the data includes around 2055 different	216

diseases classified by the International Classification of Diseases (ICD-10), which were	217

also examined for recurrence.	218

**Table 3.** Data profile from the RegulaRN Leitos Gerais

Features	Values	Outcomes Discharge: 13.549

Age	≥ 60	18.641

Death: 5.092

*<* 60	19.382	Discharge: 17.860

Death: 1.522

Sex

EUP Score

SOFA

requested bed type

entry bed type

output bed type

Discharge: 16.941

Death: 3.384

| Masculine | 20.235 |
| --- | --- |
| Feminine | 17.698 |
| 2 | 17.572 |
| 3 | 7.279 |
| 4 | 5.468 |
| 5 | 5.485 |
| 6 | 1.765 |
| 7 | 367 |
| 8 | 87 |
| 1 | 29.699 |
| 2 | 6.816 |
| 3 | 1.179 |
| 4 | 329 |
| Ward | 23.863 |
| ICU | 14.160 |
| Ward | 23.858 |
| ICU | 14.165 |
| Ward | 28.137 |
| ICU | 9.886 |
| Health unit 1 | 1.234 |
| Health unit 2 | 794 |
| Health unit 3 | 299 |
| Health unit 4 | 225 |
| Health unit 5 | 367 |
| Health unit 6 | 2.104 |
| Health unit 7 | 2.865 |

Discharge: 14.468

Death: 3.230 Discharge:16.668 Death: 904

Discharge: 6.176

Death: 1.103 Discharge:4.113 Death: 1.355

Discharge: 3.543 Death:1.942 Discharge: 770

Death: 995

Discharge: 120

Death: 247 Discharge:19 Death: 68

Discharge: 26.070

Death: 3.629

Discharge: 4.652

Death: 2.164

Discharge: 598

Death: 581

Discharge: 89

Death: 240

Discharge: 21.878

Death: 1.985

Discharge: 9.531

Death: 4.629

Discharge: 21.851

Death: 2.007

Discharge: 9.558 Death:4.607 Discharge: 26.233

Death: 1.904

Discharge: 5.176

Death: 4.710

Discharge: 1.061

Death: 173

Discharge: 554

Death: 240

Discharge: 213 Death:86 Discharge: 154

Death: 71

Discharge: 227 Death:140 Discharge: 1.722

Death: 382

Discharge: 2.341

Death: 524

July 4, 2024	8/21

Provider health unit

Discharge: 1.971

| Health unit 8 | 2.331 |
| --- | --- |
| Health unit 9 | 112 |
| Health unit 10 | 8 |
| Health unit 11 | 2.138 |
| Health unit 12 | 703 |
| Health unit 13 | 101 |
| Health unit 14 | 11 |
| Health unit 15 | 422 |
| Health unit 16 | 94 |
| Health unit 17 | 5 |
| Health unit 18 | 385 |
| Health unit 19 | 703 |
| Health unit 20 | 253 |
| Health unit 21 | 2.825 |
| Health unit 22 | 1.000 |
| Health unit 23 | 586 |
| Health unit 24 | 739 |
| Health unit 25 | 737 |
| Health unit 26 | 34 |
| Health unit 27 | 396 |
| Health unit 28 | 5.192 |
| Health unit 29 | 740 |
| Health unit 30 | 1.000 |
| Health unit 31 | 598 |
| Health unit 32 | 2.183 |
| Health unit 33 | 395 |

Death: 360

Discharge: 104

Death: 8

Discharge: 7

Death: 1

Discharge: 2.094

Death: 44

Discharge: 625

Death: 78

Discharge: 56

Death: 45

Discharge: 11

Death: 0

Discharge: 225 Death:197 Discharge: 86

Death: 8

Discharge: 3

Death: 2 Discharge:233 Death:152 Discharge:703 Death:0 Discharge:211 Death:42 Discharge:2.646 Death:179 Discharge:778 Death:222 Discharge:373 Death:213 Discharge:608 Death:131 Discharge:631 Death:106 Discharge:23 Death:11 Discharge:173 Death:223 Discharge:4.320 Death:872 Discharge:662 Death:78 Discharge:850 Death:150 Discharge:438 Death:160 Discharge:1.590 Death:593 Discharge:240 Death:155

Length of stay

Health unit 34	443	Discharge:233

Death:210

Health unit 35	193	Discharge:193

Death:0

Health unit 36	4.181	Discharge:3.774

Death:407

Health unit 37	422	Discharge:422

Death:0

Health unit 38	12	Discharge:12

Death:0

Health unit 39	522	Discharge:467

Death:55

Health unit 40	161	Discharge:119

Death:42

Health unit 41	510	Discharge:256

Death:254

*<* 7	16.693	Discharge: 13.658 Death:3.035

7	LoS	14	11.499	Discharge: 9.824

≤	≤

Death: 1.675

*>*14	9.831	Discharge: 7.927

Death: 1.904

Outcomes	Discharge	31.409

Death	6.614

219

As for the statistical profile, the average age is 53.38 years, with a standard deviation	220

of 26.82 years and a median of 59 years. The average hospitalization time was 12.96	221

days, with a standard deviation of 17.67 days and a median of 7 days. The mean EUP	222

score was 3.15 and the median was 3. The mean SOFA scale was 1.2 and the median 1.	223

Regarding the ICD, Table 4 shows the ten most recurrent ICDs, followed by the	224

municipalities with the highest incidence and the hospitals that treat the most. The	225

state capital, the city of Natal, has the highest number of inhabitants and has the	226

highest incidence of ICDs 6 and 10. In contrast, Mossor´o, the second largest	227

municipality in terms of inhabitants, has a higher incidence in four of the ten. The	228

noteworthy point is that there is no significant number of requests for these diseases	229

among Parnamirim, S˜ao Gon¸calo do Amarante, and Maca´ıba municipalities, which are	230

the 3rd, 4th and 5th most populous municipalities.	231

SOFA and EUP are two tools used to evaluate the hospital’s bed priority for each	232

patient, considering that EUP revolves around SOFA, The Charlson Comorbidity Index	233

(CCI) and the Clinical Frailty Scale (CFS). According to the data, it is possible to	234

identify that EUP, has a more normalized aggregation to the outcomes classification.	235

Meanwhile, SOFA = 1, was responsible for characterizing 78% of the data, a similar	236

percentage is presented in the sum of requests with EUP 2 (46.2%), 3 (19.1%), and 4	237

(14.3%). In other words, while SOFA indicates that 78% of referrals had the same	238

degree of priority, EUP structures the same percentage into three different categories.	239

Given the health sector’s peculiarities, the EUP is an indicator that minimizes the	240

generalization of different clinical conditions.	241

Another important point to evaluate is the ICD that most frequently resulted in	242

death. Naturally, each ICD has its own intrinsic lethality level, meaning that some	243

diseases kill more than others. However, it is necessary to analyze the frequency of	244

certain occurrences and whether the incidence is local and already expected by public	245

**Table 2.** Selection of hyperparameters and values for each model.

## Models Hyperparameter Range and best values

criterion	gini or entropy;

Decision Tree

Random Forest

Gradient Boosting

Adaboost

XGBoost

MLP SGD MLP ADAM

max depth of the tree	[10, 50, 100]; min samples leaf	range [1,2,3,4];

max features	[sqrt, log2].

criterion	gini or entropy; max depth of the tree	[10, 50, 100];

number estimators	[100, 200, 400]; max features	[sqrt, log2].

criterion	friedman mse or squared error; max depth	[10, 50, 100];

number estimators	[10, 50, 100]; max features	[sqrt, log2].

learning rate	[0.1, 0.5, 1.0]

number estimators	[100, 200, 400] algorithm	[samme, samme.r]

learning rate	[0.1, 0.5, 1.0]

number estimators	[100, 200, 400]

max depth	[10, 50, 100]

colsample by tree	[0.1, 0.5, 1.0]

hidden layer sizes	[5, 25, 70] activation	tanh or relu

batch size	[16, 32, 64]

hidden layer sizes	[5, 25, 70] activation	tanh or relu

batch size	[16, 32, 64]

health institutions. Hence, with the data in hand, public health authorities can evaluate	246

and orchestrate future intervention proposals. As shown in Table 5, Unspecified	247

Pneumonia (J18.9) was the disease with the highest frequency (see Table 4) and	248

resulted in the most deaths. Around 24.5% of the patients classified with this disease	249

died, resulting in 15.9% of the total number of deaths. However, Unspecified Septicemia	250

is one of the most lethal diseases and is responsible for the death of 50.5% of patients	251

diagnosed with this disease.	252

Regarding the data correlation, shown in Fig 2, Phik’s correlation revealed that the	253

features that relate most closely to the outcome are the output bed type, requested bed	254

type, entry bed type, SOFA scale, ICD, age, EUP score, and provider unit. Length of	255

stay and gender did not present any relevant correlation for this topic.	256

## Fig 2. Presentation of the Phik correlation for RegulaRN Leitos Gerais data.

**Machine learning model results**	257

Table 2 shows the selection of hyperparameters that indicated the best results for the	258

selected models. The Decision Tree model obtained the best criterion results when the	259

entropy node division strategy was selected, max depth of the tree with a value of 50,	260

min samples leaf with a value of 1 and max feature, square root. Random Forest	261

obtained the best results with entropy (criterion), 50 (max depth of tree), 400 (number	262

estimators) and sqrt (max features). For Gradient Boosting, squared error (criterion),	263

**Table 4.** Distribution of the most frequent ICDs by municipality and hospital.

Code	Name	Frequency	Municipality with the highest incidence

Natal: 1437

Hospitals that treat the most Provider Hospital 28: 504

J18.9	Unspecified Pneumonia	4284

I21.9	Unspecified Acute Myocardial Infarction	1738

Stroke, Not Specified as Hemorrhagic

Mossor´o: 763

Santo Antˆonio: 278

Mossor´o 781

Natal: 150 Currais Novos:97 Mossor´o: 1010

Provider Hospital 6: 344

Provider Hospital 11: 333

Provider Hospital 36: 910

Provider Hospital 6: 158

Provider Hospital 7: 119

Provider Hospital 28: 969

I64

N39.0

or Ischemic	^1540^

Urinary Tract Infection of Unspecified	_965_

Localization

|  | | | Parnamirim: 79 | Provider Hospital 30: 67 |
| --- | --- | --- | --- | --- |
|  |  |  | Natal: 243 | Provider Hospital 6: 134 |
| I50.0 | Congestive Heart Failure | 905 | Mossor´o: 196 | Provider Hospital 28: 73 |
|  |  |  | Currais Novos: 97 | Provider Hospital 21: 68 |
|  |  |  | Mossor´o: 531 | Provider Hospital 36: 529 |
| I20.0 | Unstable Angina | 677 | Natal: 53 | Provider Hospital 6: 30 |
|  |  |  | Currais Novos: 8 | Provider Hospital 21: 13 |
|  |  |  | Natal: 292 | Provider Hospital 21: 293 |
| F20.8 | Other Schizophrenias | 587 | Parnamirim: 50 | Provider Hospital 7: 272 |
|  |  |  | Cear´a-Mirim: 34 | Provider Hospital 6: 9 |
|  |  |  | Natal: 180 | Provider Hospital 21: 85 |
| A46 | Erysipelas | 546 | Mossor´o: 70 | Provider Hospital 32: 64 |
|  |  |  | Caic´o: 62 | Provider Hospital 39: 33 |
|  |  |  | Natal: 151 | Provider Hospital 32: 81 |
| A41.9 | Unspecified Septicemia | 542 | Mossor´o: 115 | Provider Hospital 28: 72 |
|  |  |  | Caic´o: 67 | Provider Hospital 6: 38 |
|  |  |  | Mossor´o: 373 | Provider Hospital 36: 374 |
| I25.2 | Old Myocardial Infarction | 510 | Natal: 31 | Provider Hospital 7: 19 |
|  |  |  | Jo˜ao Cˆamara: 19 | Provider Hospital 25: 17 |

Caic´o: 150

Natal: 148

Natal: 408

Mossor´o: 83

Provider Hospital 32: 164

Provider Hospital 6: 73

Provider Hospital 21: 125

Provider Hospital 41: 102

**Table 5.** Distribution of the ICDs with the highest number of deaths

| **Code** | **Name** | **Frequency** | **Deaths/Total incidence** |
| --- | --- | --- | --- |
| J18.9 | Unspecified Pneumonia | 1052 | 24.5% |
| A41.9 | Unspecified Septicemia | 279 | 50.5% |
| I50.0 | Congestive Heart Failure | 247 | 27.3% |
| J18.0 | Unspecified Bronchopneumonia | 110 | 24.8% |
| I21.9 | Unspecified Acute Myocardial Infarction | 94 | 12.6% |

10 (max depth of tree), 50 (number estimators) and sqrt (max features). In the Adabost 264 model, the best results were: 1.0 (learning rate), 400 (number estimators), and samme.r 265 (algorithm). In XGBoost: 0.1 (learning rate), 200 (number estimators), 50 (max depth)	266 and 1.0 (colsample by tree). The MLP models used the same hyperparameters in SGD	267 and ADAM (hidden layer sizes, activation, batch size) which resulted in the same values: 268 70, relu and 32.	269

As for the results obtained by the selected metrics, XGBoost scored highest in	270

accuracy (87.77%) and recall (87.77%). On the other hand, the Random Forest model	271

(87.85%) was the most accurate, i.e. being the model that best classifies the positive	272

outcome. As for the F1-Score value, the Gradient Boosting model had the highest value	273

(87.56%). As for specificity, a parameter that assesses the classification performance of	274

the negative outcome, it can be seen that the multilayer perceptron models outperform	275

the others. The highest score was obtained by the SGD (82.94%). Table 6 shows all the	276

values obtained.	277

**Table 6.** Metrics obtained by the computer models

| Models | Accuracy | Precision | Recall | F1-Score | Specificity |
| --- | --- | --- | --- | --- | --- |
| Decision Tree | 82.97(+0.13) | 84.26(+0.19) | 82.96(+0.13) | 83.51(+0.14) | 64.36(+0.42) |
| Random Forest | 87.20(+0.01) | **87.85(+0.03)** | 87.20(+0.01) | 87.47(+0.01) | 72.98(+0.17) |
| Gradient Boosting | 87.14(+0.05) | 88.21(+0.03) | 87.14(+0.05) | **87.56(+0.03)** | 75.47(+0.24) |
| Adaboost | 86.69(+0.05) | 87.76(+0.05) | 86.69(+0.05) | 87.12(+0.05) | 74.25(+0.06) |
| XGBoost | **87.77(+0.07)** | 87.46(0.04) | **87.77(+0.07)** | 87.60(+0.10) | 66.96(+0.04) |
| MLP SGD | 83.36(+0.17) | 88.10(+0.07) | 83.36(+0.17) | 84.73(0.13) | **82.94(+0.50** |
| MLP ADAM | 82.88(+0.76) | 87.87(+0.13) | 82.88(+0.76) | 84.33(+0.61) | 82.58(+0.62) |

Based on the results of the models, we performed a chi-square statistical validation	278

to analyze whether the behavior of the models has statistical significance. For this, a	279

contingency table was created with the distribution of real and predicted values of all	280

models and for all cases a p value ¡ 0.01 was obtained.	281

As for the features that were important for training the models, the most relevant	282

features for classifying the Decision Tree were *bed type*, *age*, *provider health unit* and *icd*.	283

The non-relevant elements were *requested bed type*, *entry bed type* and *SOFA*. In the	284

Random Forest model, *output bed type*, *age*, *EUP*, *provider health unit* and *icd* scored	285

the highest, while *sex* and *SOFA* were the least relevant characteristics. For the	286

Gradient Boosting classifier *output bed type*, *age*, *EUP* and *provider health unit* were the	287

most relevant, while *sex*, *requested bed type*, *entry bed type* were the lowest scorers.	288

Adaboost considered the best characteristics to be *length of stay*, *provider health unit*,	289

*EUP* and *age*, while the least relevant were *sex*, *requested bed type* and *entry bed type*.	290

XGBoost considered *output bed type*, *EUP* and *entry bed type* as the most important	291

characteristics and *sex*, *SOFA* and *requested bed type* as the least important. For the	292

models that used MLP, Adam considered *output bed type*, *requested bed type*, *provider*	293

*health unit* and *age* to be the most relevant, while SGD considered *output bed type*,	294

*provider health unit*, *age* and *ICD* to be the most significant. The least important	295

features were *entry bed type* and *sex* for Adam; and *sex* and *requested bed type* for SGD.	296

Fig 3 and Fig 4 show the important features of the machine learning models.	297

Compared to Phik’s correlation, except for Adabost, all the other classifiers included	298

*output bed type* as the most important feature in the classification process, which	299

corroborates Phik’s correlation (*output bed* being the feature with the highest	300

correlation with the outcome) and the weaker correlation, *sex* was identified as the least	301

relevant feature for Random Forest, Grandient Boosting, Adaboost, XGBoost and SGD,	302

while *length of stay*, which is another feature that has been shown to have a low	303

correlation with the outcome, was not identified as worse in any of the classifiers,	304

however, for Adaboost this feature was the most significant.	305

## Fig 3. Feature importance of the machine learning models.

## Fig 4. Feature importance of MLP models.

The ROC curve (receiver operating characteristic curve) helps to visualize the	306

performance of classifiers to select an appropriate operating point or decision	307

threshold [53]. The discriminative capacity is usually quantified by the area under the	308

AUC curve when considering the prediction of a binary event. It relates the variation in	309

the rate of true positives and false positives predicted by the models, with results on a	310

scale of 0 to 1. Although there is no definitive consensus in the literature, most studies	311

using this tool consider an AUC between 0.7 and 0.8 to be good and acceptable, and	312

between 0.8 and 0.9 to be very good [54, 55].	313

Thus, the Decision Tree (AUC = 0.738), XGBoost (AUC = 0.766) and Random	314

Forest (AUC = 0.785) models performed well, while the Adaboost (AUC = 0.804),	315

Adam (AUC = 0.814) and SGD (AUC = 0.821) models performed better, falling into	316

the very good category. Fig 5 shows the results obtained.	317

## Fig 5. ROC curve and AUC value of all models.

**Discussion**	318

The use of artificial intelligence and computational methods to solve and predict	319

problems in the health field has been going on for some years now, and although there	320

is a considerable range of solutions in various segments, from predicting diseases by	321

diagnosing medical images [56–60] to the classification of important markers for the	322

prediction of cardiological [25, 61], and ophthalmological diseases [62, 63] or the analysis	323

of data to predict early-stage cancer [64–66]; as well as robotic mechanisms for surgery,	324

for example [67–70]. There are still some sectors that have not been explored or that	325

have made negligible contributions [57, 71–73].	326

According to Yu, Beam and Kohane [57], the association of artificial intelligence will	327

be able to contribute even more effectively to clinical practices and health management.	328

In this way, healthcare professionals will be able to reduce the time spent on repetitive	329

tasks in order to explore better treatments and clinical solutions aimed at patient care,	330

something that machines cannot do and which require more humanized treatment.	331

According to Valentim et al [8], the use of digital health solutions based on artificial	332

intelligence are already considered relevant tools by healthcare managers, as they help	333

to make decision-making more timely, effective and based on robust scientific evidence.	334

In the process of regulating hospital beds, the use of artificial intelligence helps to	335

reduce medical subjectivity in the face of the repetitive process of countless daily	336

regulations, tasks that can often become a tiring activity throughout the day. This	337

certainly contributes to minimizing errors in the indication of hospital beds, especially	338

when it comes to public health, since the daily volume of care is extremely high, as is	339

the case in the state of Rio Grande Norte in Brazil, which has a population of	340

approximately 3.5 million inhabitants. This could result in better resolutions for	341

patients, as well as better equity in access to the resources available in the public health	342

system. All of this will lead to a more timely hospitalization process for patients, and	343

consequently to better performance in terms of hospital bed turnover - better average	344

occupancy time for hospital beds across the entire public health network [2]. In general,	345

the use of machine learning tools can optimize the care process, increasing efficacy,	346

efficiency and effectiveness, which induces better resilience of the health system,	347

especially in times of crisis, as was the case during COVID-19 [8, 74, 75].	348

At the management level, in response to a more efficient regulatory system, financial	349

and human resources can be distributed in a way that is more coherent with the needs	350

of the different health sectors, promoting greater equity and better cost-effectiveness.	351

This is because having an efficient regulation process helps to minimize hospitalization	352

times and, consequently, hospital revenue costs [5, 8].	353

In this study, machine learning techniques were used in different tree and ensemble	354

models, as well as artificial neural network models on hospital bed regulation data, and	355

the aim was to classify the outcome of patients regardless of their ICD, to help the	356

regulating doctor and reduce subjectivity during the hospital bed regulation process.	357

As for the results of the computer models, XGBoost showed the best accuracy	358

(87.77%) and recall (87.77%) values, i.e. of all the models used, it classifies the data	359

better in general, regardless of the outcome (discharge or death), as well as, given the	360

positive outcome, the proportion that was correctly classified. As for the accuracy	361

indicator, which identifies which proportion of positive outcomes was correct, Random	362

Forest performed best (87.05%). As for the F1-Score, Gradient Boosting has a better	363

harmonic mean between precision and recall, i.e. it has a better balance in the metrics	364

that assess the positive outcome. Regarding specificity, a metric that assesses the	365

classification of the negative outcome, the neural network models showed the best results	366

when compared to the tree and ensemble models, achieving scores of 82.58% (ADAM)	367

and 82.94% (SGD). For the ROC-AUC, the SGD and ADAM models also performed	368

better, because, as they had a more balanced classification of positive and negative	369

outcomes, the ROC-AUC value was in the range of 82.13% and 81.42%, respectively.	370

Considering these results, the models used in this experiment are not only able to	371

predict which patients are more likely to be discharged or die, but also allow us to	372

understand which samples are being better classified concerning the outcome and the	373

best type of hospital bed according to the clinical conditions of each patient. And so,	374

the main metric analyzed should not only be accuracy; the other metrics that point to a	375

positive outcome (precision, recall and ROC-AUC) should also be maximized [2].	376

Furthermore, it also has a positive impact on the pace of work of the regulatory	377

professional, given that in situations of high demand and overload of requests, the	378

assertiveness of the regulatory process can be compromised, and so the models	379

contribute to better regulatory conduct [76, 77].	380

**Conclusion**	381

This study used the regulation database of the *RegulaRN Leitos Gerais* platform	382

between 2021 and 2024 in machine learning models to predict the outcome of discharge	383

and death in different diseases that require hospitalization. The results of this article	384

show that there is no single model that obtains the best accuracy, precision, recall,	385

F1-Score, specificity, and ROC-AUC metrics. Thus, depending on the objectives of the	386

regulatory professionals, it should be observed which model can provide the best result	387

based on the desired metric, i.e. for example, if the regulator’s objective is to observe	388

the best classifications for the positive outcome, it should use XGBoost and Random	389

Forest; If the objective is to evaluate the best classification for the negative outcome,	390

the multilayer perceptron models should be evaluated.	391

It should be noted that artificial intelligence computer models enhance the activities	392

carried out in the healthcare and management sectors. Research in this area should	393

therefore be increasingly explored in order to minimize the precariousness and	394

weaknesses that exist in the different health segments. In this way, this research also	395

aims to make a positive contribution to the health system such as the SUS, which aims	396

to guarantee universal and comprehensive access to health with equity.	397

The study’s limitations include the fact that the health professionals did not provide	398

more precise information on some of the data that could have been better analyzed in	399

the models, such as whether the patient was pregnant. During the evaluation of the	400

database, some gaps were found in the data, which is why it was not included for	401

training the models. However, for some diseases, knowing whether the patient is	402

pregnant or not and the appropriate length of pregnancy are essential. In addition, this	403

study considered the same evaluation of hospital outcomes in different diseases with	404

different morbidity scales. For future work, creating a new feature that can categorize	405

diseases by morbidity could contribute to a more appropriate classification of the	406

models. Furthermore, trying to identify which treatment protocols were used to treat	407

certain diseases can also be a relevant indicator for classifying models.	408

**Funding**	409

The present study was funded through the Project Regula SESAP-RN/FUNCERN,	410

grant number 69/2021, carried out by the Laboratory of Technological Innovation in	411

Health (LAIS) of the Federal University of Rio Grande do Norte (UFRN) in cooperation	412

with the Secretary of Public Health of Rio Grande do Norte.	413

# Acknowledgments 414

We would like to thank the Public Health Secretariat of Rio Grande do Norte	415

(SESAP/RN), the Health Technological Innovation Laboratory (LAIS) of the Federal	416

University of Rio Grande do Norte (UFRN), the Advanced Innovation Center (NAVI) of	417

the Federal Institute do Rio Grande do Norte (IFRN), to LyRIDS, ECE-Engineering	418

School and the Department of Informatics and Applied Mathematics for the support	419

necessary for the development of this research.	420

**References**	421

1. Bastos LBR, Barbosa MA, Rosso CFW, Oliveira LMdAC, Ferreira IP, Bastos	422

DAdS, et al. Practices and challenges on coordinating the Brazilian Unified	423

Health System. Revista de Sau´de Pu´blica. 2020;54:25.	424

2. Barreto TdO, Veras NVR, Cardoso PH, Fernandes FRdS, Medeiros LPdS,	425

Bezerra MV, et al. Artificial intelligence applied to analyzes during the pandemic:	426

COVID-19 beds occupancy in the state of Rio Grande do Norte, Brazil. Frontiers	427

in Artificial Intelligence. 2023;6:1290022.	428

3. Brasil. Portaria nº 1.559, de 1º de agosto de 2008. Institui a Pol´ıtica Nacional de	429

Regula¸c˜ao do Sistema U´nico de Sau´de-SUS. Di´ario Oficial da Uni˜ao. 2008;.	430

4. Brasil. Portaria nº 2, de 28º de setembro de 2017. Consolidac¸a˜o das normas sobre	431

as pol´ıticas nacionais de Sau´ do Sistema U´nico de Sau´de-SUS. Di´ario Oficial da	432

Uni˜ao. 2017;.	433

5. Maldonado RN, Savio RO, Feij´o VBER, Aroni P, Rossaneis MA, Haddad	434

MdCFL. Hospital indicators after implementation of bed regulation strategies: an	435

integrative review. Revista Brasileira de Enfermagem. 2021;74:e20200022.	436

6. Cordeiro MF. SISREG: uma ferramenta de desafios e avanc¸os para a garantia do	437

direito a sau´de. 2015;.	438

7. Junior JRM, de Souza Junior AA, da Luz AEdJ. The impact of COVID-19 on	439

the municipal Regulation System (SISREG) of Rio de Janeiro (RJ). Research,	440

Society and Development. 2024;13(4):e5613445564–e5613445564.	441

8. Valentim RAdM, Lima TS, Cortez LR, Barros DMdS, Silva RDd, Paiva JCd,	442

et al. The relevance a technology ecosystem in the Brazilian National Health	443

Service’s Covid-19 response: the case of Rio Grande do Norte, Brazil. Ciˆencia &	444

Sau´de Coletiva. 2021;26:2035–2052.	445

9. Medina MVB. An´alise da utiliza¸c˜ao da Escala Quick Sequential Organ Failure	446

Assessment para tomada de decisa˜o na regulac¸a˜o de leitos de UTI. Universidade	447

Federal do Rio Grande do Norte; 2023.	448

10. Konder M, O’Dwyer G. Regulation of access to hospital beds in emergency care	449

and the development of integrated health services. 2019;.	450

11. Kim M, Lee JY, Park JS, Kim HA, Hyun M, Suh YS, et al. Lessons from a	451

COVID-19 hospital, Republic of Korea. Bulletin of the World Health	452

Organization. 2020;98(12):842.	453

12. Lu Y, Guan Y, Zhong X, Fishe JN, Hogan T. Hospital beds planning and	454

admission control policies for COVID-19 pandemic: A hybrid computer	455

simulation approach. In: 2021 IEEE 17th International Conference on	456

Automation SCience and Engineering (CASE). IEEE; 2021. p. 956–961.	457

13. Pecoraro F, Luzi D, Clemente F. The efficiency in the ordinary hospital bed	458

management: A comparative analysis in four European countries before the	459

COVID-19 outbreak. Plos one. 2021;16(3):e0248867.	460

14. Shi F, Li H, Liu R, Liu Y, Liu X, Wen H, et al. Emergency preparedness and	461

management of mobile cabin hospitals in China during the COVID-19 pandemic.	462

Frontiers in Public Health. 2022;9:763723.	463

15. Alavinejad M, Mellado B, Asgary A, Mbada M, Mathaha T, Lieberman B, et al.	464

Management of hospital beds and ventilators in the Gauteng province, South	465

Africa, during the COVID-19 pandemic. PLOS global public health.	466

2022;2(11):e0001113.	467

16. Kuzior A, Kashcha M, Kuzmenko O, Lyeonov S, Broz˙ek P. Public health system	468

economic efficiency and COVID-19 resilience: Frontier DEA analysis.	469

International Journal of Environmental Research and Public Health.	470

2022;19(22):14727.	471

17. Taylor CA, Draney MT, Ku JP, Parker D, Steele BN, Wang K, et al. Predictive	472

medicine: computational techniques in therapeutic decision-making. Computer	473

Aided Surgery: Official Journal of the International Society for Computer Aided	474

Surgery (ISCAS). 1999;4(5):231–247.	475

18. Shahid N, Rappon T, Berta W. Applications of artificial neural networks in	476

health care organizational decision-making: A scoping review. PloS one.	477

2019;14(2):e0212356.	478

19. Tian S, Yang W, Le Grange JM, Wang P, Huang W, Ye Z. Smart healthcare:	479

making medical care more intelligent. Global Health Journal. 2019;3(3):62–65.	480

20. Panagiotou OA, H¨ogg LH, Hricak H, Khleif SN, Levy MA, Magnus D, et al.	481

Clinical application of computational methods in precision oncology: a review.	482

JAMA oncology. 2020;6(8):1282–1286.	483

21. Bian J, Modave F. The rapid growth of intelligent systems in health and health	484

care; 2020.	485

22. Gupta PK, Ramachandran AT, Keerthi AM, Dave PS, Giridhar S, Kallapur SS,	486

et al. An overview of clinical decision support system (CDSS) as a computational	487

tool and its applications in public health. Applications in ubiquitous computing.	488

2021; p. 81–117.	489

23. Moulaei K, Shanbehzadeh M, Mohammadi-Taghiabad Z, Kazemi-Arpanahi H.	490

Comparing machine learning algorithms for predicting COVID-19 mortality.	491

BMC medical informatics and decision making. 2022;22(1):2.	492

24. Albuquerque G, Fernandes F, Barbalho IM, Barros DM, Morais PS, Morais AH,	493

et al. Computational methods applied to syphilis: where are we, and where are	494

we going? Frontiers in Public Health. 2023;11:1201725.	495

25. Carvalho DRd, Arau´jo BGd, Lacerda JMT, Dantas MdCR, H´ekis HR, Valentim	496

RAdM. An architecture for online transient detection in electrocardiogram	497

signals on the MP-HA protocol. Revista Brasileira de Engenharia Biom´edica.	498

2012;28:346–354.	499

26. Levantesi S, Pizzorusso V. Application of machine learning to mortality modeling	500

and forecasting. Risks. 2019;7(1):26.	501

27. Shamout F, Zhu T, Clifton DA. Machine learning for clinical outcome prediction.	502

IEEE reviews in Biomedical Engineering. 2020;14:116–126.	503

28. Huang Y, Talwar A, Chatterjee S, Aparasu RR. Application of machine learning	504

in predicting hospital readmissions: a scoping review of the literature. BMC	505

medical research methodology. 2021;21:1–14.	506

29. Dixit RR. Risk Assessment for Hospital Readmissions: Insights from Machine	507

Learning Algorithms. Sage Science Review of Applied Machine Learning.	508

2021;4(2):1–15.	509

30. Iwase S, Nakada Ta, Shimada T, Oami T, Shimazui T, Takahashi N, et al.	510

Prediction algorithm for ICU mortality and length of stay using machine learning.	511

Scientific reports. 2022;12(1):12912.	512

31. Baak M, Koopman R, Snoek H, Klous S. A new correlation coefficient between	513

categorical, ordinal and interval variables with Pearson characteristics.	514

Computational Statistics & Data Analysis. 2020;152:107043.	515

32. Iwendi C, Bashir AK, Peshkar A, Sujatha R, Chatterjee JM, Pasupuleti S, et al.	516

COVID-19 patient health prediction using boosted random forest algorithm.	517

Frontiers in public health. 2020;8:357.	518

33. Aljameel SS, Khan IU, Aslam N, Aljabri M, Alsulmi ES. Machine	519

Learning-Based Model to Predict the Disease Severity and Outcome in	520

COVID-19 Patients. Scientific programming. 2021;2021(1):5587188.	521

34. Endo PT, Santos GL, de Lima Xavier ME, Nascimento Campos GR, de Lima LC,	522

Silva I, et al. Illusion of truth: analysing and classifying COVID-19 fake news in	523

brazilian portuguese language. Big Data and Cognitive Computing. 2022;6(2):36.	524

35. Chawla NV, Bowyer KW, Hall LO, Kegelmeyer WP. SMOTE: synthetic minority	525

over-sampling technique. Journal of artificial intelligence research.	526

2002;16:321–357.	527

36. Maiga J, Hungilo GG, et al. Comparison of machine learning models in	528

prediction of cardiovascular disease using health record data. In: 2019	529

international conference on informatics, multimedia, cyber and information	530

system (ICIMCIS). IEEE; 2019. p. 45–48.	531

37. Hamida S, El Gannour O, Cherradi B, Ouajji H, Raihani A. Optimization of	532

machine learning algorithms hyper-parameters for improving the prediction of	533

patients infected with COVID-19. In: 2020 ieee 2nd international conference on	534

electronics, control, optimization and computer science (icecocs). IEEE; 2020. p.	535

1–6.	536

38. Papaiz F, Dourado Jr MET, de Medeiros Valentim RA, Pinto R, de Morais AHF,	537

Arrais JP. Ensemble-imbalance-based classification for amyotrophic lateral	538

sclerosis prognostic prediction: identifying short-survival patients at diagnosis.	539

BMC Medical Informatics and Decision Making. 2024;24(1):80.	540

39. Divya KS, Bhargavi P, Jyothi S. Machine learning algorithms in big data	541

analytics. Int J Comput Sci Eng. 2018;6(1):63–70.	542

40. Liu J, Wang L, Zhang L, Zhang Z, Zhang S. Predictive analytics for blood	543

glucose concentration: an empirical study using the tree-based ensemble	544

approach. Library Hi Tech. 2020;38(4):835–858.	545

41. Rahul K, Banyal RK, Goswami P, Kumar V. Machine learning algorithms for big	546

data analytics. In: Computational Methods and Data Engineering: Proceedings	547

of ICMDE 2020, Volume 1. Springer; 2021. p. 359–367.	548

42. Charbuty B, Abdulazeez A. Classification based on decision tree algorithm for	549

machine learning. Journal of Applied Science and Technology Trends.	550

2021;2(01):20–28.	551

43. Rigatti SJ. Random forest. Journal of Insurance Medicine. 2017;47(1):31–39.	552
44. Fafalios S, Charonyktakis P, Tsamardinos I. Gradient boosting trees. Gnosis	553

Data Analysis PC. 2020;1.	554

45. Schapire RE. Empirical inference. Berlin, Heidelberg. 2013; p. 37–52.	555
46. Sheng C, Yu H. An optimized prediction algorithm based on XGBoost. In: 2022	556

International Conference on Networking and Network Applications (NaNA).	557

IEEE; 2022. p. 1–6.	558

47. Popescu MC, Balas VE, Perescu-Popescu L, Mastorakis N. Multilayer perceptron	559

and neural networks. WSEAS Transactions on Circuits and Systems.	560

2009;8(7):579–588.	561

48. Kingma DP, Ba J. Adam: A method for stochastic optimization. arXiv preprint	562

arXiv:14126980. 2014;.	563

49. Pedregosa F. Scikit-learn: Machine learning in python Fabian. Journal of	564

machine learning research. 2011;12:2825.	565

50. Ensor KB, Glynn PW. Stochastic optimization via grid search. Lectures in	566

Applied Mathematics-American Mathematical Society. 1997;33:89–100.	567

51. Bergstra J, Bengio Y. Random search for hyper-parameter optimization. Journal	568

of machine learning research. 2012;13(2).	569

52. Ahsan MM, E Alam T, Trafalis T, Huebner P. Deep MLP-CNN model using	570

mixed-data to distinguish between COVID-19 and Non-COVID-19 patients.	571

Symmetry. 2020;12(9):1526.	572

53. Bradley AP. The use of the area under the ROC curve in the evaluation of	573

machine learning algorithms. Pattern recognition. 1997;30(7):1145–1159.	574

54. De Hond AA, Steyerberg EW, Van Calster B. Interpreting area under the receiver	575

operating characteristic curve. The Lancet Digital Health. 2022;4(12):e853–e855.	576

55. Nahm FS. Receiver operating characteristic curve: overview and practical use for	577

clinicians. Korean journal of anesthesiology. 2022;75(1):25–36.	578

56. Mohammad-Rahimi H, Nadimi M, Ghalyanchi-Langeroudi A, Taheri M,	579

Ghafouri-Fard S. Application of machine learning in diagnosis of COVID-19	580

through X-ray and CT images: a scoping review. Frontiers in cardiovascular	581

medicine. 2021;8:638011.	582

57. Yu KH, Beam AL, Kohane IS. Artificial intelligence in healthcare. Nature	583

biomedical engineering. 2018;2(10):719–731.	584

58. Davenport T, Kalakota R. The potential for artificial intelligence in healthcare.	585

Future healthcare journal. 2019;6(2):94–98.	586

59. Owoyemi A, Owoyemi J, Osiyemi A, Boyd A. Artificial Intelligence for	587

Healthcare in Africa. Frontiers in digital health 2: 6; 2020.	588

60. Yang CC. Explainable artificial intelligence for predictive modeling in healthcare.	589

Journal of healthcare informatics research. 2022;6(2):228–239.	590

61. Cuocolo R, Perillo T, De Rosa E, Ugga L, Petretta M. Current applications of	591

big data and machine learning in cardiology. Journal of geriatric cardiology: JGC.	592

2019;16(8):601.	593

62. Barros DM, Moura JC, Freire CR, Taleb AC, Valentim RA, Morais PS. Machine	594

learning applied to retinal image processing for glaucoma detection: review and	595

perspective. Biomedical engineering online. 2020;19:1–21.	596

63. Srivastava O, Tennant M, Grewal P, Rubin U, Seamone M. Artificial intelligence	597

and machine learning in ophthalmology: A review. Indian Journal of	598

Ophthalmology. 2023;71(1):11–17.	599

64. Kourou K, Exarchos TP, Exarchos KP, Karamouzis MV, Fotiadis DI. Machine	600

learning applications in cancer prognosis and prediction. Computational and	601

structural biotechnology journal. 2015;13:8–17.	602

65. Firmino M, Angelo G, Morais H, Dantas MR, Valentim R. Computer-aided	603

detection (CADe) and diagnosis (CADx) system for lung cancer with likelihood	604

of malignancy. Biomedical engineering online. 2016;15:1–17.	605

66. Galvao-Lima L, Morais H, Valentim R, Barreto E. miRNAs as biomarkers for	606

early cancer detection and their application in the development of new diagnostic	607

tools. Biomedical engineering online. 2021;20:1–21.	608

67. Zhao B, Waterman R, Urman R, Gabriel RA. A machine learning approach to 609 predicting case duration for robot-assisted surgery. Journal of Medical Systems. 610 2019;43:1–32.	611
68. Panesar S, Cagle Y, Chander D, Morey J, Fernandez-Miranda J, Kliot M.	612

Artificial intelligence and the future of surgical robotics. Annals of surgery.	613

2019;270(2):223–226.	614

69. Zhou XY, Guo Y, Shen M, Yang GZ. Application of artificial intelligence in	615

surgery. Frontiers of medicine. 2020;14:417–430.	616

70. Moglia A, Georgiou K, Georgiou E, Satava RM, Cuschieri A. A systematic review	617

on artificial intelligence in robot-assisted surgery. International Journal of	618

Surgery. 2021;95:106151.	619

71. Fernandes YYMP, Arau´jo GTd, Arau´jo BGd, Dantas MdCR, Carvalho DRd,	620

Valentim RAdM. ILITIA: telehealth architecture for high-risk gestation	621

classification. Research on Biomedical Engineering. 2017;33(3):237–246.	622

72. Reddy S. Explainability and artificial intelligence in medicine. The Lancet	623

Digital Health. 2022;4(4):e214–e215.	624

73. Schwalbe N, Wahl B. Artificial intelligence and the future of global health. The	625

Lancet. 2020;395(10236):1579–1586.	626

74. Ammar W, Kdouh O, Hammoud R, Hamadeh R, Harb H, Ammar Z, et al.	627

Health system resilience: Lebanon and the Syrian refugee crisis. Journal of global	628

health. 2016;6(2).	629

75. Massuda A, Hone T, Leles FAG, De Castro MC, Atun R. The Brazilian health	630

system at crossroads: progress, crisis and resilience. BMJ global health.	631

2018;3(4):e000829.	632

76. Muhammad L, Algehyne EA, Usman SS, Ahmad A, Chakraborty C, Mohammed	633

IA. Supervised machine learning models for prediction of COVID-19 infection	634

using epidemiology dataset. SN computer science. 2021;2(1):1–13.	635

77. Silva Junior CL, Guabiraba KPdL, Gomes GG, Andrade CLTd, Melo EA.	636

Outpatient regulation in Primary Care in the municipality of Rio de Janeiro,	637

Brazil, based on the local regulatory doctors. Ciˆencia & Sau´de Coletiva.	638

2022;27:2481–2493.	639
